# Ecdysteroids as Potent Enzyme Inhibitors and Verification of Their Activity Using In Vitro and In Silico Docking Studies

**DOI:** 10.3390/life12060824

**Published:** 2022-05-31

**Authors:** Nilufar Z. Mamadalieva, Hidayat Hussain, Adriano Mollica, Gokhan Zengin, Rano Z. Mamadalieva, Sameh S. Elhady, Sana A. Fadil, Mohamed L. Ashour, Fadia S. Youssef

**Affiliations:** 1 Institute of the Chemistry of Plant Substances, Academy Sciences of Uzbekistan, Tashkent 100170, Uzbekistan; 2 Department of Bioorganic Chemistry, Leibniz Institute of Plant Biochemistry, Weinberg 3, 06120 Halle, Germany; hidayat.hussain@ipb-halle.de; 3 Department of Pharmacy, University “G. d’Annunzio” of Chieti-Pescara, 66100 Chieti, Italy; a.mollica@unich.it; 4 Department of Biology, Science Faculty, Selcuk University, Konya 42130, Turkey; gokhanzengin@selcuk.edu.tr; 5 Kokand State Pedagogical Institute, Turon Str. 23, Kokand 713000, Uzbekistan; rmamadalieva@yahoo.com; 6 Department of Natural Products, Faculty of Pharmacy, King Abdulaziz University, Jeddah 21589, Saudi Arabia; ssahmed@kau.edu.sa (S.S.E.); safadil@kau.edu.sa (S.A.F.); 7 Department of Pharmaceutical Sciences, Pharmacy Program, Batterjee Medical College, P.O. Box 6231, Jeddah 21442, Saudi Arabia; 8 Department of Pharmacognosy, Faculty of Pharmacy, Ain Shams University, Cairo 11566, Egypt; fadiayoussef@pharma.asu.edu.eg

**Keywords:** α-amylase, ADMET, cholinesterase, chemometrics, ecdysteroids, tyrosinase, molecular docking, drug discovery, health care

## Abstract

Ecdysteroids represent arthropods’ steroidal hormones, and they exist in about 5–6% of plant species. In this study, the enzyme inhibitory activity of 20 ecdysteroids was assessed for the first time via determining their inhibition versus acetylcholinesterase, butyrylcholinesterase, tyrosinase, as well as α-amylase enzymes. Furthermore, 20-Hydroxyecdysone-2,3,22-tri-*O*-acetate (**4**) showed the highest inhibition of acetylcholinesterase and butyrylcholinesterase with values of 5.56 and 4.76 mg GALAE/g, respectively. All ecdysteroids displayed tyrosinase inhibitory effects, whereas the most potent was viticosterone E (**7**) with 78.88 mg KAE/g. Most ecdysteroids had similar amylase inhibitory properties; meanwhile, the best α-amylase inhibitory potential was observed with viticosterone E-diacetonide (**18**) (0.35 mmol ACAE/g). Most of the tested compounds showed tyrosinase inhibitory potential; therefore, they were exposed to molecular docking evaluation using the tyrosinase enzyme. Viticosterone E (**7**) showed the best ranking score with a docking score of −5.716 Kcal/mol and made three separate H-bonds with Gly281, Asn81, and His85. From ADMET /TOPKAT in silico evaluation, it was obvious that most of the compounds displayed reasonable pharmacodynamic and pharmacokinetic properties; however, their toxicity should be carefully monitored by adjusting their doses while investigating their activity after incorporation into dosage forms. Principal component analysis (PCA) based upon the in vitro and in silico data was carried out to visualize the differences between the tested compounds better. PCA score plot successfully classifies the compounds into four main clusters that, in turn, reflects the similarities and differences among the clustered compounds with respect to their biological, pharmacokinetic, and pharmacodynamic properties that are mainly influenced by the similarity in the chemical structure. Thus, ecdysteroids can act as effective drug entities for alleviating several disorders owing to their enzyme inhibitory potential.

## 1. Introduction

Nowadays, drug discovery based on natural products is felt mandatory worldwide owing to their efficacy and relative safety compared to synthetic drugs. Plant-derived drug entities have shown remarkable therapeutic effects in ameliorating many human ailments and are strongly appreciated by a large category of patients owing to their natural origin [1]. The pronounced effects of plant materials are mainly attributed to their richness by secondary metabolites comprising flavonoids, triterpenes, volatile oils, anthraquinones, as well as tannins [2].

Besides, enzyme inhibitors are defined as compounds that particularly target the enzyme’s active sites via binding with its functional moieties, consequently leading to a decrease in the rate of enzymatic reaction [3]. They have been recognized as a new strategy for combating many diseases such as diabetes, Alzheimer’s, cancer, hypertension, and many other disorders [4]. It is worth highlighting that those cholinesterase inhibitors are greatly adopted to alleviate dementia comprising Alzheimer’s disease, which is described as an irreversible neurological state that happens continuously, and its occurrence is increased with age [5]. Meanwhile, tyrosinase is necessary for the production of melanin, which causes hyperpigmentation, and thus its inhibition could regulate dark skin patches [6]. Furthermore, α-amylase is crucial in the process of carbohydrate digestion causing blood glucose elevation, and hence its inhibition could effectively decrease postprandial glucose level and regulate postprandial hyperglycemia in diabetic patients [7].

Ecdysteroids represent the arthropods’ steroidal hormones that play a pivotal role in controlling their metamorphosis, reproduction, molting, and diapause in addition to playing similar roles in other invertebrates’ phyla [8]. Ecdysteroids exist in ≈5–6% of plant species with higher concentrations than their concentrations in arthropods, where they are believed to contribute to the deterrence of invertebrate predators [9]. Basically, 20-hydroxyecdysone is the most common and predominant ecdysteroid in both arthropods and plants; meanwhile, a wide array of structural analogues has been explored, particularly from plant origin. Although the activity of most ecdysteroids has not been fully investigated, some ecdysteroids reveal certain significant biological potential, such as s elicitors of novel gene-switch systems and stimulating protein synthesis and reducing its decreasing protein catabolism and thus increasing the muscle mass [10,11].

Herein, our main target was to investigate the enzyme inhibitory activity of 20 ecdysteroids for the first time via assessing their inhibition versus acetylcholinesterase, butyrylcholinesterase, tyrosinase, as well as α-amylase enzymes. Meanwhile, the tested ecdysteroids were exposed to molecular docking evaluation on tyrosinase enzyme. Moreover, the postulation of ADMET (absorption, distribution, metabolism, excretion, and toxicity) characteristics as well as TOPKAT (toxicity prediction) for all of the tested entities was carried out using Discovery Studio 4.5 software (Accelrys Inc., San Diego, CA, USA). Additionally, chemometrics was performed using unsupervised pattern recognition represented by principal component analysis (PCA) based on the in vitro and in silico data to better visualize the differences among the tested compounds and classify them according to their similarities and differences in biological, pharmacodynamic, and pharmacokinetic potential.

## 2. Materials and Methods

### 2.1. Selection of the Tested Ecdysteroids

Ecdysteroids and their derivatives (**1**–**20**) selected for this study were obtained from the Institute of the Chemistry of Plant Substances (Tashkent, Uzbekistan) with purity percentages > 95%. They were 20-hydroxyecdysone (**1**), 20-hydroxyecdysone-22-benzoate (**2**), 20-hydroxyecdysone-2,3,22,25-tetraacetate (**3**), 20-hydroxyecdysone-2,3,22-tri-O-acetate (**4**), turkesterone (**5**), 2-deoxy-20-hydroxyecdysone (**6**), viticosterone E (**7**), integristerone A (**8**), polypodine B (**9**), ecdysone (**10**), 2-deoxyecdysone (**11**), ecdysone-2,3-di-O-acetate (**12**), ecdysone-22-O-acetate (**13**), 26-hydroxypolypodine B (**14**), 26-hydroxypolypodine B-2,3,22,26-tetraacetate (**15**), 20-hydroxyecdysone-20,22-acetonide (**16**), 20-hydroxyecdysone-2,3;20,22-diacetonide (**17**), viticosterone E-diacetonide (**18**), cyasterone (**19**), and 22-O-acetyl cyasterone (**20**).

### 2.2. Determination of the Enzyme Inhibitory Potential of the Selected Ecdysteroids by In Vitro Assays

#### 2.2.1. Cholinesterase (ChE) Inhibitory Activity

ChE inhibitory activity was performed by adopting Ellman’s method, as previously reported by Aktumsek et al. accompanied by performing slight modifications [12]. Briefly, in a 96-well microplate, 50 µL of the examined samples was added to 125 µL of DTNB and 25 µL of acetyl (AChE) or butyrylcholinesterase (BChE) in 25 µL of Tris–HCl buffer (pH 8.0) and subsequently incubated for 15 min at 25 °C. The reaction was initiated by adding 25 µL of acetylthiocholine iodide or butyrylthiocholine chloride. In the same manner, the blank was prepared by mixing the samples with the previously mentioned reagents without adding the enzyme solutions (AChE or BChE). The absorbance samples and blank were recorded at 405 nm after being incubated for 10 min at 25 °C, followed by subtraction of the blank absorbance from the sample. The results were expressed as galantamine equivalents (mg GALAE/g) [13,14]. The assays were carried out in triplicate, and the differences in the results of the tested samples were assessed by ANOVA assays (Tukey’s test).

#### 2.2.2. Tyrosinase Inhibitory Activity 

Tyrosinase inhibitory activity was evaluated by performing slight modification in dopachrome assay, employing l-DOPA as a substrate, as previously reported [15]. Basically, in a 96-well microplate, 25 µL of the examined samples was added to 40 µL of tyrosinase solution as well as 100 µL of phosphate buffer (pH 6.8) and subsequently incubated for 15 min at 25 °C. The reaction was started by the addition of 40 µL of l-DOPA. In the same manner, the blank was prepared by mixing the samples with the previously mentioned reagents without adding the enzyme solution. The absorbance samples and blank were recorded at 492 nm after being incubated for 10 min at 25 °C, followed by subtraction of the blank absorbance from the sample. The results were expressed as kojic acid equivalents (mg KAE/g). The assays were performed in triplicate, and the differences in the extracts were evaluated by ANOVA assays (Tukey’s test).

#### 2.2.3. α-Amylase Inhibitory Activity

The α-Amylase inhibitory potential was assessed by the Caraway–Somogyi iodine/potassium iodide (IKI) assay as previously described by Lazarova et al. (2015) after certain modifications [16]. In brief, in a 96-well microplate, 25 µL of the examined samples was added to 50 µL of α-amylase solution as well as 100 µL of phosphate buffer (pH 6.9) supplemented with sodium chloride (6 mM) and subsequently incubated for 10 min at 37 °C. The reaction was stopped by adding 1 mM of HCl (25 µL) and iodine–potassium iodide (100 µL) solution. In the same manner, the blank was prepared by mixing the samples with the previously mentioned reagents without adding the enzyme solution. The absorbance samples and blank were recorded at 630 nm, followed by subtraction of the blank absorbance from the sample. The results were expressed as acarbose equivalents (mg ACAE/g). The assays were performed in triplicate, and the differences in the extracts were evaluated by ANOVA assays (Tukey’s test).

#### 2.2.4. Statistical Analysis 

All experiments were conducted in triplicate, and expression of the results was as mean ± SD. One-way variance analysis (ANOVA) was used to detect the differences among examined compounds, followed by Tukey’s honest significant difference post hoc test with =0.05 using the SPSS version 14.0 program. Construction of the graphs was performed by GraphPad Prism 5 (GraphPad Software, Inc., San Diego, CA, USA).

### 2.3. Molecular Modelling Study

#### 2.3.1. Preparation of the Receptor

The docking studies were performed for compounds **3**, **4**, **7**, **12**, and **16** using mushroom tyrosinase (PDB ID 2Y9X) in complex with its inhibitor tropolone. It has 2.3 Å resolution and it is the first crystal structure of the full fungal tyrosinase complex; thus, it has been selected for its similarity with the enzyme used for the biological assays in this work. [17]. The aforementioned compounds have been selected among the others because they have shown the most relevant inhibitory activity toward tyrosinase in the assays performed in this work, as reported in Table 1. The hydrogens state assignment of the enzyme’s crystal structure was performed at pH 7.4 by the PROpKa method implemented in the Protein Preparation Wizard panel included in Maestro, employing the well-established procedure commonly adopted by our research group, e.g., [18], then the hydrogens of the enzymes were subjected to minimization by force field OPLS3 [19]. Preparation of the targeted enzyme for the docking experiment was achieved by polishing the raw crystallographic enzyme file using the PrepWizard module embedded in Maestro Schrödinger [20] that mainly removes the non-catalytic water as well as the additional molecules existing in the PDB file.

#### 2.3.2. Preparation of the Ligands

The docking experiment was performed on the five most active compounds on tyrosinase assays, compounds **3**, **4**, **7**, **12**, and **16** that belong to ecdysteroids. Preparation of the tested ligands that were manually drawn by the Maestro 3D builder tool starting from the structure of viticosterone E as a template, available online at the PubChem database—was made using the LigPrep tool embedded in Maestro 2017-1 [20] prior to the molecular docking, followed by neutralization at pH 7.4 by Epik as well as minimization by force field OPLS3 [13].

#### 2.3.3. Molecular Docking

The docking experiments were conducted using Maestro 2017 software [20] with the scoring function XP employed previously to dock secondary bioactive metabolites to tyrosinase [13,21]. The grid for docking was automatically assigned via centering the box on the crystallographic inhibitor, extending around the ligand center with a box side of 25 Å. The docking scores obtained for ecdysteroids docked to tyrosinase were then determined. The enzymatic pocket of the crystallized enzyme contains a binuclear copper-binding site in the deoxy-state, in which three histidines coordinate each copper ion. The side chains of these histidines have their orientation fixed by hydrogen bonds or, in the case of His85, by a thioether bridge with the side chain of Cys83. The specific tyrosinase inhibitor tropolone forms a pre-Michaelis complex with the enzyme. It binds near the binuclear copper site without directly coordinating the copper ions [17]. 

### 2.4. Evaluation of ADMET/TOPKAT Properties

The selected ecdysteroids were exposed to ADMET evaluation (absorption, distribution, metabolism, excretion, and toxicity) as well as toxicity postulation (TOPKAT) by Discovery Studio 2016 (Accelrys Inc., San Diego, CA, USA) to evaluate their pharmacodynamic, pharmacokinetic, as well as toxicity properties. Human intestinal absorption (HIA), aqueous solubility, blood–brain barrier penetration (BBB), plasma protein binding prediction (PPB), hepatotoxicity level, and cytochrome P450 2D6 inhibition were taken as ADMET descriptors. However, carcinogenic impact on male and female rat FDA, Ames mutagenicity, rat oral LD50, and rat chronic LOAEL (lowest observed adverse effect level) in addition to the ocular and dermal irritant effect were selected as TOPKAT parameters [22,23].

### 2.5. Multivariate Data Analysis

Multivariate data analysis was carried out using the data obtained from different in vitro biological assays, and the data resulting from ADMET postulation representing human intestinal absorption, aqueous solubility, blood–brain barrier penetration (BBB), hepatotoxicity, plasma protein binding (PPB), in addition to cytochrome P450 (2D6) inhibition. This was performed using the unsupervised pattern recognition technique by principal component analysis (PCA) through CAMO’s Unscrambler^®^ X 10.4 software (Computer-Aided Modeling, As, Norway) [24,25].

## 3. Results and Discussion

### 3.1. Ecdysteroids Selected in this Study 

**Figure 1:**
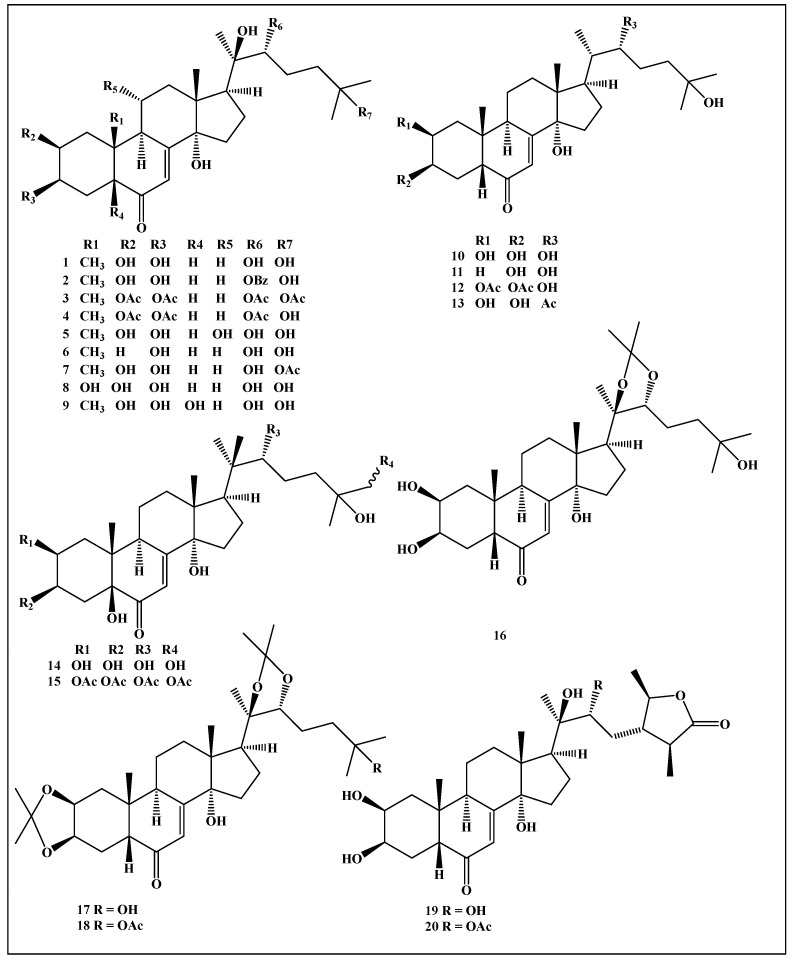
Chemical structures of selected ecdysteroids used in the current study.

Little information was traced in the literature regarding the enzyme inhibitory properties of ecdysteroids [26]. Thus, the reported results shed light on the use of the enzyme inhibitory properties of ecdysteroids in pharmaceutical and medical applications. A total of 20 ecdysteroids (**1**–**20**) were selected in the study, and they are sketched in Figure 1.

### 3.2. Determination of the Enzyme Inhibitory Potential of the Ecdysteroids by In Vitro Assays 

#### 3.2.1. Cholinesterase (ChE) Inhibitory Activity

**Figure 2:**
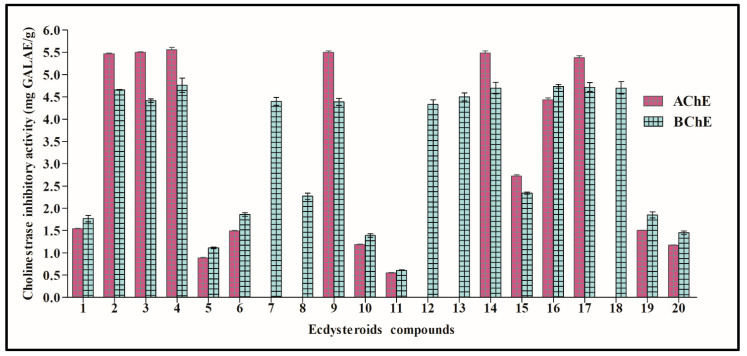
Cholinestrase inhibitory potential of 20 selected ecdysteroids expressed in mg GALAE/g. Values are represented as mean ± S.D.; GALAE: Galatamine equivalent.

The enzyme inhibitory effects of tested ecdysteroids were examined against acetylcholinesterase (AChE) and butyrylcholinesterase (BChE) as well. The inhibition of AChE and BChE is closely related to managing memory functions in Alzheimer’s disease. Their inhibition increases the level of acetylcholine in the synaptic gap, which improves cognitive capacity in Alzheimer’s disease. Several natural compounds have exhibited remarkable cholinesterase inhibition abilities, and indeed, many works have been carried out for this purpose [27]. The results in Figure 2 reveal that the most effective compound was 20-hydroxyecdysone-2,3,22-tri-*O*-acetate (**4**) (5.56 mg GALAE/g) for AChE, followed by 20-hydroxyecdysone-2,3,22,25-tetraacetate (**3**) (5.51 mg GALAE), polypodine B (**9**) (5.50 mg GALAE/g), and 26-hydroxypolypodine B (**14**) (5.49 mg GALAE/g). However, the five compounds viticosterone E (**7**), integristerone A (**8**), ecdysone-2,3-di-*O*-acetate (**12**), ecdysone-22-*O*-acetate (**13**), and viticosterone E-diacetonide (**18**) revealed no activity on AChE. Concerning BChE, similar to AChE, the best inhibitory effect was provided by 20-hydroxyecdysone-2,3,22-tri-*O*-acetate (**4**) (4.76 mg GALAE/g). In addition, four compounds, 26-hydroxypolypodine B (**14**), 20-hydroxyecdysone-20,22-acetonide (**16**), 20-hydroxyecdysone-2,3;20,22-diacetonide (**17**), and viticosterone E-diacetonide (**18**), showed similar BChE inhibitory effects (*p* > 0.05). Thus, it can be concluded that ecdysteroids could effectively alleviate Alzheimer’s disease via the prohibition of cholinesterases. This fact was also supported by an earlier study, which reported that the 20-hydroxyecdysone derivative (septanoecdysone) from *Atriplex portulacoides* roots exhibited significant AChE inhibitory effects [28]. In addition, three ecdysteroids had both AChE and BChE inhibitory effects. These observations may highlight a need for further studies to explain the cholinesterase inhibitory properties of ecdysteroids in our previous work [26]. 

#### 3.2.2. Tyrosinase Inhibitory Activity

Tyrosinase is a key enzyme in melanin synthesis, and its inhibition could manage hyperpigmentation problems such as hyperpigmented patches or spots. It is a multifunctional oxidase enzyme that is involved in the hydroxylation of tyrosine and oxidation to dopaquinone. Several compounds, such as kojic acid and hydroquinone derivatives, have been used as tyrosinase inhibitors in the cosmeceutical industries [29]. The results in Table 1 show that all ecdysteroids displayed tyrosinase inhibitory effects, whereas the most potent was viticosterone E (**7**) with 78.88 mg KAE/g. In addition, 20-hydroxyecdysone-2,3,22,25-tetraacetate (**3**), 20-hydroxyecdysone-2,3,22-tri-*O*-acetate (**4**), and 20-hydroxyecdysone-20,22-acetonide (**16**) showed significant tyrosinase inhibitory effects but the activities were statistically similar (*p* > 0.05). Thus, from the presented results, it is obvious that the tested ecdysteroids could be incorporated in many cosmeceutical natural preparations targeting skin disorders, such as skin-whitening products.

#### 3.2.3. α-Amylase Inhibitory Activity

Diabetes mellitus is an aggressive metabolic disease characterized by polyphagia, hyperglycemia, polydipsia, as well as frequent urination that influences about 10% of the population worldwide [23]. Amylase is a key enzyme in starch hydrolysis, and this is the main target for controlling blood sugar levels in diabetes patients [30]. In the present study, most ecdysteroids had similar amylase inhibitory properties (*p* > 0.05); meanwhile, the best α-amylase inhibitory potential was observed with viticosterone E-diacetonide (**18**) (0.35 mmol ACAE/g). Results showing the inhibitory effect of all the examined ecdysteroids versus α-amylase are presented in Table 1. Hence, ecdysteroids can be incorporated into various formulations to alleviate hyperglycemia and ameliorate high blood glucose levels.

### 3.3. Molecular Modelling Studies 

**Figure 3:**
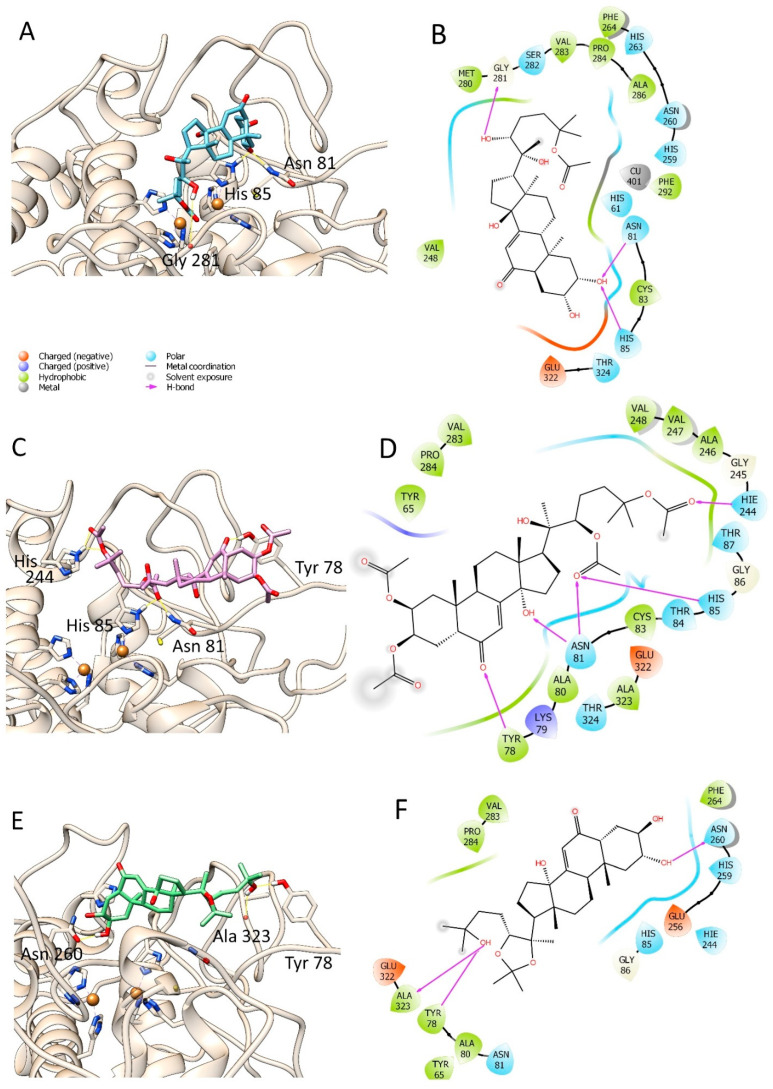
Docking poses of viticosterone E (**7**) (**A**,**B**), 20-hydroxyecdysone-2,3,22,25-tetraacetate (**3**) (**C**,**D**), and 20-hydroxyecdysone-20,22-acetonide (**16**) (**E**,**F**).

**Figure 4:**
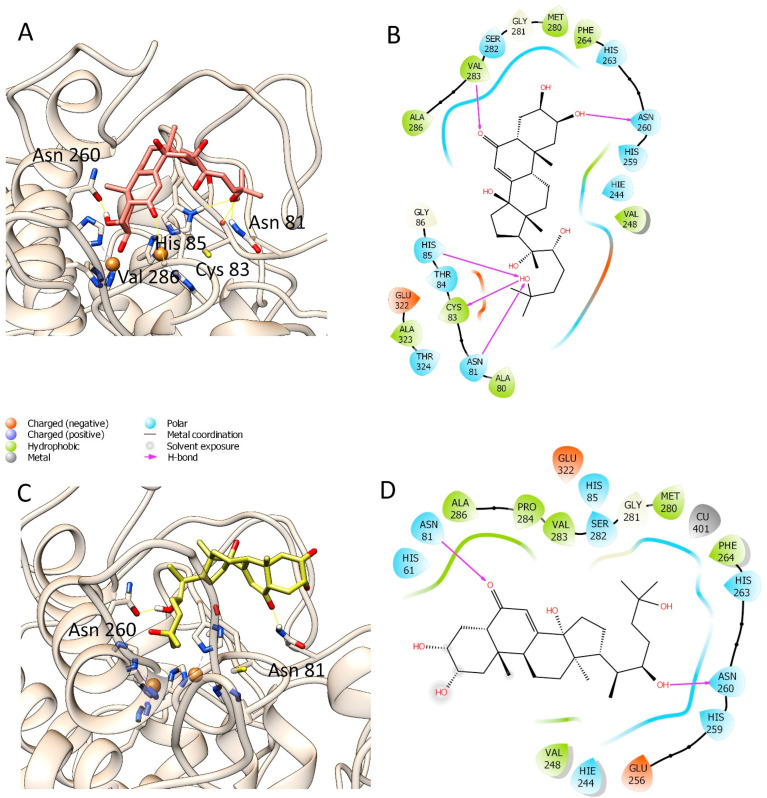
Docking poses of 20-hydroxyecdysone-2,3,22-tri-*O*-acetate (**4**) (**A**,**B**) and Ecdysone-2,3-di-*O*-acetate (**12**) (**C**,**D**).

Since we detected significant tyrosinase inhibitory properties in the tested compounds, we investigated the interactions between compounds and tyrosinase in molecular modelling studies. The interactions between each selected ligand and the tyrosinase have been visualized using Maestro 2017-1 [20] and depicted through UCSF-Chimera (Figure 3 and Figure 4), and their docking scores are recorded in Table 2. It was observed that compounds **7** and **3** have the best docking score among the selected compounds. Compound **7** showed the best ranking score with a docking score of −5.716 Kcal/mol and made three H-bonds with Gly281, Asn81, and His85, respectively, as displayed in Figure 3A,B. Compound **3** was the second best compound, also able to establish several interactions with tyrosinase (docking score = −5.451 Kcal/mol); in the details, two hydrogen bonds with Asn81, and one hydrogen bond each with His85 and Tyr78 (Figure 3C,D). Meanwhile, compounds **4** and **16** resulted in a good docking score (−5.636 Kcal/mol and −5.375 Kcal/mol, respectively) and interacted with tyrosinase by forming several hydrogen bonds with Asn260, Val283, Asn81, Cys83, and His85 for compound **4** and Asn260, Ala2, and Tyr78 for compound **16**. However, compound **12** showed the lowest docking score (docking score = −4.840 Kcal/mol,) where it can make one H-bond with Asn260 and Asn81. It is worth noting that none of the selected compounds could strongly bind to the copper atoms present deep inside the enzymatic cavity. On the contrary, they can bind the key residues of histidine 85, which is involved in the coordination of the catalytic metals. However, compound **7** showed in the best ranked docking pose a distance between one hydroxyl group and one Cu atom of the enzyme less than four angstroms. Thus, this compound may also interact with this key atom. The obtained docking poses were submitted to the free energy estimation by the MM-GBSA method of the prime module in Maestro to investigate further the binding energy of the best poses [13]. These results are summarized in Table 2. Among the selected substances, only compounds **3** and **7** have good and similar binding energy of −31.91 Kcal/mol and −29.26 Kcal/mol, respectively, followed by compound **16** (−24.75 Kcal/mol) and compound **12** (−20.67 Kcal/mol).

The obtained computational data may explain the capability of the selected substance to dock at the enzymatic cavity of tyrosinase and exert an inhibitory activity, as found experimentally in this work. The inhibitory effects of the ecdysone derivatives mentioned above against tyrosinase have not been widely investigated; therefore, their anti-tyrosinase activity is not well defined in the literature. A previous study by our team [26] on 20-hydroxyecdysone (**1**) extracted from *Silene viridiflora*, together with 2-deoxy-20-hydroxyecdysone (**6**) and 2-deoxyecdysone (**11**), reported a modest inhibitory activity in comparison with the standard tyrosinase inhibitor kojic acid (20.34  ±  0.39 mg KAE/g of 2-deoxy-20-hydroxyecdysone and 19.65  ±  2.78 mg KAE/g of 20-hydroxyecdysone). Hence, it can be concluded that ecdysteroids displayed a potential low to moderate inhibitory activity against tyrosinase, but further studies could be necessary to understand in depth their biological role.

### 3.4. Evaluation of ADMET/TOPKAT Properties

**Figure 5:**
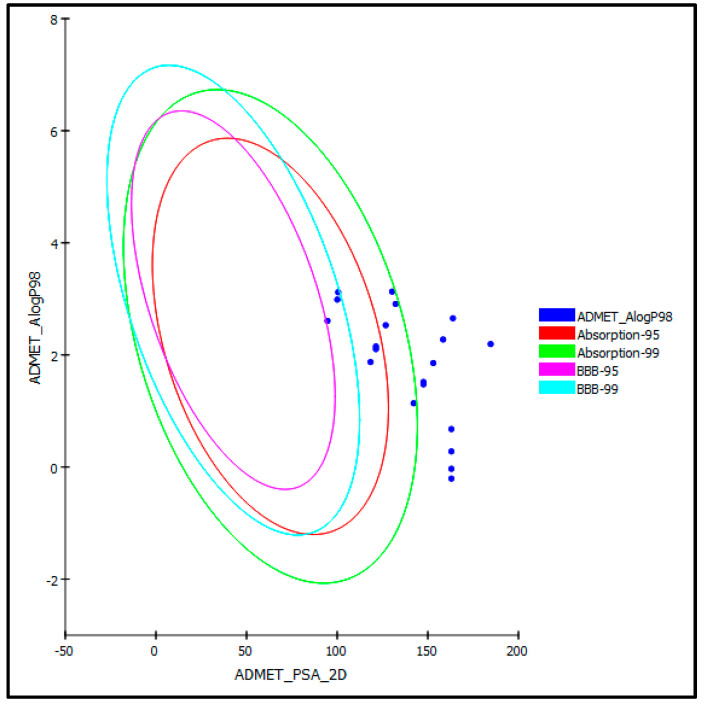
ADMET plot for selected ecdysteroids showing 95% and 99% confidence limit ellipses corresponding to the blood–brain barrier (BBB) and the human intestinal absorption models in ADMET_AlogP98.

Determination of ADMET/TOPKAT characteristics was performed to outline the pharmacodynamic, the pharmacokinetics, and the toxicity potential of the examined ecdysteroids. Regarding human intestinal absorption level, half of the examined compounds comprising 20-hydroxyecdysone (**1**), 20-hydroxyecdysone-22-benzoate (**2**), 2-deoxy-20-hydroxyecdysone (**6**), ecdysone (**10**), 2-deoxyecdysone (**11**), ecdysone-2,3-di-*O*-acetate (**12**), ecdysone-22-*O*-acetate (**13**), 20-hydroxyecdysone-20,22-acetonide (**16**), 20-hydroxyecdysone-2,3;20,22-diacetonide (**17**), and viticosterone E-diacetonide (**18**) revealed good to moderate absorption levels. Thus, they lie inside the 95% and 99% absorption ellipses, as illustrated in the ADMET plot (Figure 5), whereas the rest of the compounds showed low to very low absorption levels. However, most of the tested ecdysteroids exhibited good to optimal solubility except for compounds **17** and **18**, which showed low solubility levels. For penetration of the blood–brain barrier (BBB), nearly all of the examined compounds showed undefined levels of penetration taking level 4. Thus, they lie outside the 99% of the BBB confidence ellipse (Figure 5) in contrast to compounds **11**, **17**, and **18** which lie within 99% of the BBB confidence ellipse, exerting low penetration of the BBB. 

Furthermore, plasma protein binding pattern (PPB) was determined for all of the compounds since the free drug concentration serves as a crucial factor during the evaluation of the probable pharmaceutical activity, and the results in Table 3 reveal that the PPB of all of the compounds is below 90%. Besides estimating the inhibitory potential of the examined compounds on Cytochrome P450 2D6 (CYP2D6), which is involved in various xenobiotics metabolism and whose prohibition may result in uncontrolled drug–drug interactions, all of the tested ecdysteroids proved to be non-inhibitors of CYP2D6. They also displayed no hepatotoxicity, as revealed in the in silico study, except compound **8**, which experienced certain toxicity (Table 3).

For the TOPKAT evaluation illustrated in Table 4, all examined ecdysteroids showed no mutagenicity regarding in silico *Ames* mutagenicity examination. Unfortunately, all compounds showed certain carcinogenicity in rat female FDA except compounds **2;** meanwhile, most of the compounds do not elicit carcinogenicity in rat male FDA except compounds **1**, **3**, **4**, **7**, **8**, **12**, **18**, and **19**. Besides, most of the compounds exhibited moderate dermal irritation except compounds **2**, **5**, **8**, and **17** that showed mild skin irritancy. Regarding ocular irritation, most of the compounds showed moderate to severe irritation in contrast to compounds **2** and **15**, which revealed no irritancy towards the eye. The examined ecdysteroids revealed rat oral LD_50_ in the range between 4.11 and 29.14 g/kg body weight, where compound **18** exerted the lowest rat oral LD_50_ compared to compound **5**, which showed the highest value. Similarly, for rat chronic LOAEL values for the compounds that range between 0.001 and 0.03 g/kg body weight, where the lowest values were displayed by compounds **17** and **18** (Table 4). From ADMET/TOPKAT in silico evaluation, it was obvious that most of the compounds displayed reasonable pharmacodynamic and pharmacokinetic properties; however, their toxicity should be carefully monitored by adjusting their doses while investigating their activity during their incorporation in dosage forms.

### 3.5. Multivariate Data Analysis

**Figure 6:**
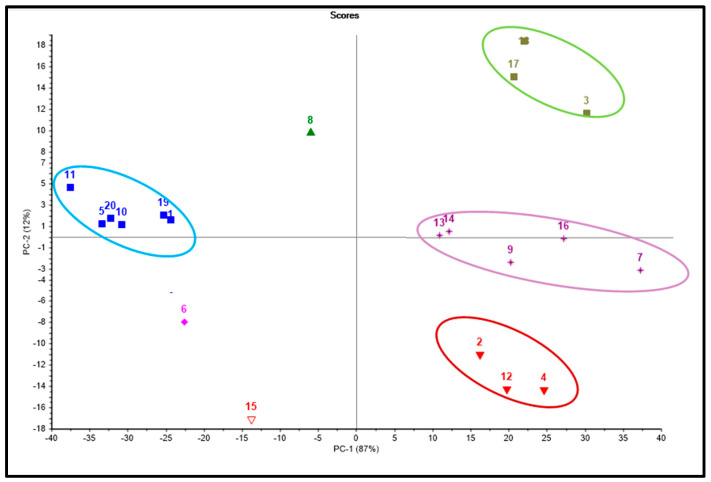
PCA score plot of different ecdysteroids employing multivariate analysis; compounds are given numbers illustrated in Figure 1.

Chemometric analysis using the unsupervised pattern recognition technique based on the in vitro results, the docking studies, as well as the ADMET evaluation were used to classify the compounds in terms of their similarity and differences via compiling the overall properties. Principal components analysis, as shown in Figure 6, revealed that both PC1 and PC2 could effectively discriminate all studied ecdysteroids, representing 87% and 21% of the total variance, respectively. The PCA score plot successfully classified the compounds into four main clusters, where three of them lie on the right-hand side of the plot, whereas one cluster lies on its left-hand side. This classification, in turn, reflects the similarity and differences among the clustered compounds in their biological behavior guided by the results of in vitro assays and their pharmacokinetic and pharmacodynamic properties mainly influenced by the similarity in the chemical structure. Values for in vitro assays, absorption levels, solubility levels, BBB penetration levels, and plasma protein binding (PPB) levels were the chosen variables that influenced the clustering of the compounds. Besides, compounds **6**, **8**, and **15** failed to cluster and are scattered in the score plot (Figure 6), reflecting their overall behavioral difference with respect to other tested compounds.

## 4. Conclusions

Plant-derived drug entities showed remarkable therapeutic effects in ameliorating many human ailments. Besides, enzyme inhibitors have been recognized as a new strategy for combating many diseases such as diabetes, Alzheimer’s, cancer, hypertension, and many other disorders. Ecdysteroids represent the arthropods’ steroidal hormones and exist in about 5–6% of plant species but with higher concentrations. In this study, the enzyme inhibitory activity of 20 ecdysteroids was assessed for the first time by determining their inhibition against acetylcholinesterase, butyrylcholinesterase, tyrosinase, as well as α-amylase enzymes. It was concluded that most of the tested ecdysteroids showed considerable enzyme inhibitory potential and thus could be used as a therapeutic strategy to treat many diseases. Additionally, from ADMET/TOPKAT in silico evaluation, it is obvious that most of the compounds displayed reasonable pharmacodynamic and pharmacokinetic properties; however, their toxicity should be carefully monitored during their incorporation in dosage forms. Chemometric analysis based on in vitro and in silico results successfully classifies the compounds into four main clusters that reflect the similarities and differences among the clustered compounds in their biological, pharmacokinetic, and pharmacodynamic properties.

## Figures and Tables

**Table 1:** Tyrosinase inhibitory potential expressed in mg KAE/g and α-amylase inhibitory activity expressed as mmol ACAE/g for selected ecdysteroids.

Compound	Tyrosinase Inhibitory Potential	α-Amylase Inhibitory Activity
**1**	19.65 ± 2.78 ^ef^	0.10 ± 0.03 ^def^
**2**	60.50 ± 4.69 ^bc^	0.16 ± 0.05 ^cd^
**3**	71.41 ± 5.79 ^ab^	0.17 ± 0.01 ^cd^
**4**	69.10 ± 4.89 ^ab^	0.14 ± 0.04 ^de^
**5**	10.67 ± 0.92 ^fg^	0.07 ± 0.01 ^ef^
**6**	20.34 ± 0.39 ^ef^	0.10 ± 0.03 ^def^
**7**	79.88 ± 1.36 ^a^	0.14 ± 0.01 ^de^
**8**	37.38 ± 0.09 ^d^	0.11 ± 0.02 ^def^
**9**	62.49 ± 5.77 ^bc^	0.14 ± 0.01 ^de^
**10**	13.30 ± 1.10 ^fg^	0.07 ± 0.01 ^ef^
**11**	6.36 ± 0.20 ^g^	0.05 ± 0.01 ^f^
**12**	64.45 ± 4.87 ^bc^	0.24 ± 0.03 ^bc^
**13**	53.19 ± 2.64 ^c^	0.16 ± 0.02 ^cd^
**14**	54.19 ± 4.86 ^c^	0.17 ± 0.04 ^cd^
**15**	31.51 ± 2.60 ^de^	0.15 ± 0.02 ^de^
**16**	69.33 ± 3.04 ^ab^	0.32 ± 0.01 ^ab^
**17**	61.59 ± 9.30 ^bc^	0.11 ± 0.01 ^def^
**18**	62.94 ± 7.44 ^bc^	0.35 ± 0.05 ^a^
**19**	18.60 ± 3.29 ^efg^	0.10 ± 0.03 ^def^
**20**	11.74 ± 1.49 ^fg^	0.09 ± 0.01 ^def^

Values are reported as mean ± S.D. GALAE: Galatamine equivalent; KAE: Kojic acid equivalent; ACAE: Acarbose equivalent; Na: Not active. Different letters indicate significant differences in the tested compounds (*p* < 0.05, by ANOVA assay).

**Table 2:** Docking scores and ΔG free binding energy of the selected ecdysteroids within the active pocket of tyrosinase enzyme expressed as kcal/mol.

Compound	XP Docking Score	ΔG Free Binding Energy
**3**	−5.451	−31.91
**4**	−5.336	−14.26
**7**	−5.716	−29.26
**12**	−4.840	−20.67
**16**	−5.375	−24.75

**Table 3:** ADMET (absorption, distribution, metabolism, excretion, and toxicity) criteria of selected ecdysteroids.

Compounds	Absorption Level	Solubility Level	BBB Level	PPB Level	CPY2D6	Hepato-Toxic	Alog *p*98	PSA-2D
**1**	1	3	4	False	NI	NT	1.14	142.193
**2**	1	3	4	False	NI	NT	2.09	130.308
**3**	3	3	4	False	NI	NT	2.65	163.855
**4**	3	3	4	False	NI	NT	2.28	158.440
**5**	3	4	4	False	NI	TOX	−0.03	163.009
**6**	0	3	4	False	NI	NT	2.10	121.378
**7**	2	3	4	False	NI	NT	1.52	147.609
**8**	3	4	4	False	NI	TOX	−0.21	163.009
**9**	3	4	4	False	NI	NT	0.28	163.009
**10**	0	3	4	False	NI	NT	2.15	121.378
**11**	0	3	3	False	NI	NT	3.12	100.562
**12**	1	3	4	False	NI	NT	2.91	132.209
**13**	1	3	4	False	NI	NT	2.53	126.793
**14**	3	4	4	False	NI	NT	0.68	163.009
**15**	3	3	4	False	NI	NT	2.19	184.67
**16**	0	3	4	False	NI	NT	1.87	118.422
**17**	0	2	3	False	NI	NT	2.61	94.652
**18**	0	2	3	False	NI	NT	2.99	100.067
**19**	2	3	4	False	NI	NT	1.48	147.609
**20**	3	3	4	False	NI	NT	1.86	153.024

0, 1, 2, and 3 indicates good, moderate, low, and very low absorption, respectively; 0, 1, 2, 3, 4, and 5 indicates extremely low, very low but possible, low, good, optimal, and too soluble, respectively; 0, 1, 2, 3, and 4 denote very high, high, medium, low, and undefined, penetration via BBB, respectively. PBB, plasma protein binding; False means less than 90%; NI: non-inhibitor; NT: non-toxic.

**Table 4:** TOPKAT evaluation of the tested of selected ecdysteroids using drug discovery software.

Compound	Ames Prediction	Rat Oral LD50	Rat Chronic LOAEL	Skin Irritancy	Ocular Irritancy	Rat Female FDA	Rat Male FDA
**1**	Non-Mutagen	21.76	0.02	Moderate	Severe	Carcinogen	Carcinogen
**2**	Non-Mutagen	17.04	0.03	Mild	None	Non-Carcinogen	Non-Carcinogen
**3**	Non-Mutagen	12.77	0.01	Moderate	Mild	Carcinogen	Carcinogen
**4**	Non-Mutagen	17.30	0.01	Moderate	Severe	Carcinogen	Carcinogen
**5**	Non-Mutagen	29.14	0.03	Mild	Severe	Carcinogen	Non-Carcinogen
**6**	Non-Mutagen	16.58	0.02	Moderate	Severe	Carcinogen	Non-Carcinogen
**7**	Non-Mutagen	24.02	0.02	Moderate	Moderate	Carcinogen	Carcinogen
**8**	Non-Mutagen	21.25	0.03	Mild	Severe	Carcinogen	Carcinogen
**9**	Non-Mutagen	13.00	0.03	Moderate	Severe	Carcinogen	Non-Carcinogen
**10**	Non-Mutagen	15.44	0.02	Moderate	Severe	Carcinogen	Non-Carcinogen
**11**	Non-Mutagen	11.95	0.01	Moderate	Severe	Carcinogen	Non-Carcinogen
**12**	Non-Mutagen	16.58	0.01	Moderate	Severe	Carcinogen	Carcinogen
**13**	Non-Mutagen	17.09	0.01	Moderate	Moderate	Carcinogen	Non-Carcinogen
**14**	Non-Mutagen	9.75	0.03	Moderate	Severe	Carcinogen	Non-Carcinogen
**15**	Non-Mutagen	6.83	0.01	Moderate	None	Carcinogen	Non-Carcinogen
**16**	Non-Mutagen	7.55	0.004	Moderate	Severe	Carcinogen	Non-Carcinogen
**17**	Non-Mutagen	4.92	0.001	Mild	Severe	Carcinogen	Non-Carcinogen
**18**	Non-Mutagen	4.11	0.001	Moderate	Moderate	Carcinogen	Carcinogen
**19**	Non-Mutagen	8.60	0.006	Moderate	Moderate	Carcinogen	Carcinogen
**20**	Non-Mutagen	6.83	0.01	Moderate	Moderate	Carcinogen	Non-Carcinogen

## Data Availability

Data are available within the article.
